# A Retrospective Comparative Study in Patients With Cocaine Use Disorder Comorbid With Attention Deficit Hyperactivity Disorder Undergoing an rTMS Protocol Treatment

**DOI:** 10.3389/fpsyt.2021.659527

**Published:** 2021-03-25

**Authors:** Stefano Cardullo, Luis J. Gómez Pérez, Diego Cuppone, Michela Sarlo, Nicola Cellini, Alberto Terraneo, Luigi Gallimberti, Graziella Madeo

**Affiliations:** ^1^Fondazione Novella Fronda, Piazza Castello, Padova, Italy; ^2^Department of Communication Sciences, Humanities and International Studies, University of Urbino Carlo Bo, Urbino, Italy; ^3^Department of General Psychology, University of Padova, Padova, Italy; ^4^Padova Neuroscience Center, University of Padova, Padova, Italy

**Keywords:** attention deficit hyperactivity disorder, cocaine use disorder, craving, repetitive transcranial magnetic stimulation, dorsolateral prefrontal cortex, dopamine

## Abstract

**Background:** Adult attention-deficit/hyperactivity disorder (ADHD) is associated with high comorbidity with other psychiatric diseases, including cocaine use disorder (CocUD). Given the common fronto-striatal dysfunction, ADHD patients often use cocaine as self-medication for ameliorating symptoms by increasing striatal dopamine release. Yet, comorbidity with ADHD is related to poor treatment outcomes. CocUD has been treated with transcranial magnetic stimulation (TMS), but no studies investigated the outcomes in patients comorbid with ADHD.

**Methods:** Twenty-two ADHD/CocUD and 208 CocUD-only participants received a high-frequency (15 Hz) rTMS treatment stimulating the left-DLPFC. We investigated whether both groups of patients shared similar demographic and clinical characteristics at baseline. Then, we monitored the effect of treatment testing for potential differences between groups.

**Results:** At baseline demographic, toxicology and clinical features were not different between the two groups except for global severity index (GSI from SCL-90): patients of ADHD/CocUD group reported higher general symptomatology compared to the CocUD-only group. Concerning the effect of treatment, both groups significantly improved over time regarding cocaine use, craving, and other negative affect symptoms. No differences were observed between groups.

**Conclusions:** To our knowledge, this is the first study comparing the demographic characterization and rTMS clinical improvements of patients with a dual diagnosis of ADHD and CocUD against CocUD-only patients. Cocaine use and common self-reported withdrawal/abstinence symptoms appear to benefit from rTMS treatment with no differences between groups. Future studies are needed to further investigate these preliminary results.

## Introduction

Attention deficit hyperactivity disorder (ADHD) is a neurobehavioral disorder characterized by a persistent pattern of inattention and/or hyperactivity-impulsivity interfering with functioning or development (1). ADHD symptomatology begins in childhood but often persists into adulthood (2), with high comorbidity rates with other mental disorders (3) such as substance use disorders (SUDs). Indeed, the prevalence of ADHD is considerably higher among individuals with SUDs than in the general population (4–14). The co-occurrence of these disorders has relevant prognostic implications, as it is associated with a more severe course of substance use, a higher rate of psychiatric comorbidity, and poorer treatment outcome (4, 5, 7, 15–19). Several studies show similar disruptions of the brain dopamine (DA) fronto-striatal system and executive control impairments in adults with ADHD (20) and in people who chronically use drugs, as cocaine (21, 22). The impairment of dopamine signaling in individuals with ADHD may explain the higher risk of taking addictive drugs, as substances of abuse acutely increase brain DA concentration, and might transitorily improve ADHD symptoms (23). Moreover, these DA dysfunctions have been linked to the initiation and maintenance of addictive behaviors (24), indicating that drug addiction represents a dramatic dysregulation of brain motivational circuits (25). This evidence has led to the development of neurobiology-based interventions to modify functions of the affected neurocircuitry (26). Repetitive transcranial magnetic stimulation (rTMS) appears a novel and promising neuromodulation approach to the treatment of SUDs (27). rTMS influences neural electrical activity at the network level by inducing either short-or long-term effects through the application of magnetic pulses (28). Long-lasting rTMS-induced changes may impact behavioral manifestations of addictive disorders as craving, intake, or relapse (29). Preliminary clinical studies have shown reductions in cocaine craving and intake after rTMS treatments (30–35). In addition, it was reported a positive effect of rTMS on other symptoms connected to substance use and deeply related to the fronto-striatal functioning (36). The modulation of relevant addiction dimensions (e.g., anhedonia) was found to play a key role in modulating the response to the rTMS treatment (37, 38). Considering the evidence of cortical disinhibition across different psychiatric conditions (39), this brain stimulation technique has shown to provide some benefits also in ADHD subjects improving the core symptoms, including attention deficits, hyperactivity/impulsivity, and oppositional defiance (40, 41). Thus, considering that ADHD comorbidity negatively affects conventional treatment results for SUDs as cocaine use disorders (CocUD) (17), the present study aimed to assess the therapeutic response in terms of substance use and accompanying withdrawal symptoms in a sample of CocUD patients with and without ADHD symptoms who underwent a high frequency rTMS stimulation protocol over the left dorsolateral prefrontal cortex (L-DLPFC).

## Methods

### Participant Selection

Two-hundred and thirty participants diagnosed as suffering from cocaine use disorder (CocUD) were recruited after they voluntarily referral to a specialty outpatient clinic, Center for Addiction in Padua (Italy). Patients signed informed consent on the day of clinic intake and agreed that their data could be used for research. Patients were informed that the data collected would be processed in accordance with the law on privacy and compliance with Legislative Decree No. 196 of June 30, 2003, “Personal Data Protection Code” ensuring anonymity. The data were extracted from patient clinical records and anonymized for analysis. All subjects gave their informed consent for inclusion before they participated in the study. This is a retrospective chart review of data from 230 patients with CocUD who were treated with an rTMS protocol from 2015 to 2019 in an open-label, no sham control study investigating sleep disturbances. The protocol, limited to the retrospective chart review, was approved by the Ethical Committee for the Psychological Research, Departments of Psychology, University of Padua (Protocol no. 3185, code 82F319362FA08A4C9498620BF072CB72), and the study was conducted in accordance with the Declaration of Helsinki. The current retrospective analysis is listed at ClinicalTrials.gov (identifier: NCT03733821).

Participants were 22 to 59 years old and met diagnostic criteria for CocUD according to the Diagnostic and Statistical Manual of Mental Disorders – 5 (DSM 5) (1), as assessed by a clinical psychiatrist specializing in substance use disorders (SUDs). Exclusion criteria included a prior history of other psychiatric diseases, including major depression, schizophrenia, bipolar disorder or other psychosis, current alcohol and other substance abuse or dependence (excluding nicotine, and caffeine), pregnancy or breastfeeding, personality disorders or sleep disturbances deemed to be the primary disease, current unstable medical illness, substantial neurological illness, and any contraindication for rTMS (including implanted metal and devices in the body, or history of epilepsy). From the entire sample of 230 participants, we identified 22 patients diagnosed as suffering from ADHD as assessed by the structured Diagnostic Interview for ADHD in adults (DIVA 2.0) (42). The clinical suspicion of adult-ADHD arises from the evidenced role of self-medication in symptom control of cocaine rather than a research of the euphoric properties of the substance. As confirmation of the diagnosis, 19 out of 22 ADHD patients were pharmacologically treated with atomoxetine (mean: 34 mg/die, range: 18–80 mg/die), in addition to the rTMS treatment, with a significant reduction of inattentive and hyperactive symptoms. Thus, we benchmarked the outcomes of the sample of 22 CocUD patients in comorbidity with ADHD against a large cohort of 208 CocUD patients. All participants were required to keep medication use stable throughout the study. During the whole period of observation, cocaine use was assessed either *via* a urine drug test, at each visit, or *via* reports from the patient or significant others. The urine drug screen panel also included the following: morphine, methadone, THC, phencyclidine, amphetamine, and methamphetamine.

### Treatment

Each patient underwent rTMS using a medical device (MagPro R30) targeting the L-DLPFC. The stimulation parameters, in accord with international recommendations for patient safety and ethics (43), were: frequency 15 Hz, intensity 100% of the motor threshold, 60 impulses per stimulation train, inter-train interval 15 s, and 40 total trains, for a session duration of 13 min. To best identify the L-DLPFC [Montreal Neurological Institute (MNI) coordinates x: −50, y: 30, z: 36], we used an optical TMS navigator (Localite, St. Augustin, Germany) and a magnetic resonance image (MRI) template. Treatment characteristics are the same described in our previous studies (30, 34): twice-daily rTMS sessions for the first five consecutive days of treatment, followed by twice-daily rTMS sessions once a week over eleven weeks. The time interval between the two sessions within each day was 45–60 min. Then, rTMS was re-administered throughout follow-up on an individualized basis to patients who reported lapses to cocaine use, and to patients whose clinical evaluations showed ongoing cocaine craving, including stress-induced craving. At each session, adverse events, including seizures, syncopes, neurological complications, or subjective complaints about memory, concentration, pain, headache, vertigo, or fatigue were assessed with a self-report questionnaire specifically developed by us for this purpose.

### Measures

The primary outcome measure was cocaine use. It was assessed through a combination of urine screening, self-report, and reports by collateral informants (typically family members). Firstly, we considered the lapse to cocaine use. In this analysis, for consistency with our previous works (30, 34), the “zero” day for follow-up monitoring was set at 8 days after the initial 5-day course of rTMS. After that 8-day grace period, any indication of cocaine use was coded as a lapse.

In addition to lapse to cocaine use during follow-up, we evaluated the categorical reduction in cocaine frequency level. We adopted a harm reduction approach already validated for alcohol and cocaine consumption (44, 45). Based on the cocaine use during the 30 days before the assessment, we specified three frequency levels at baseline and day 90: abstinence, low-frequency use (one to 4 days of cocaine use in the past month), and high-frequency use (5 or more days of cocaine use in the past month). We also created a “change” variable to indicate a variation in cocaine frequency level from baseline to day 90: increase one level, no change, decrease one level, decrease two levels.

Secondary outcome measures were craving, perceived sleep quality, depression, anxiety, and other negative affect symptoms, assessed with the following scales: Cocaine Craving Questionnaire (CCQ) (46), Pittsburgh Sleep Quality Index (PSQI) (47), Beck Depression Inventory–II (BDI-II) (48), Self-rating Anxiety Scale (SAS) (49), and Symptoms checklist 90 - Revised (SCL-90-R) (50). Participants were assessed at baseline, immediately after completion of the first week of treatment (Day 5), and 30, 60, and 90 days after the beginning of treatment (Day 30–Day 60–Day 90). The instructions of BDI-II require the participant to consider the last 2 weeks preceding the test; thus, it was not included in the assessment on Day 5. Several participants did not complete every scale at every time point, for the main following reasons: clinical response, missing follow-up visit, missing TMS session, and refusal.

### Statistical Analyses

Independent sample *t*-tests and chi-squares were performed to evaluate differences in the demographic and clinical characterization of patients at baseline.

Concerning the treatment primary outcomes, we used Kaplan- Meier survival analysis to calculate the median number of days until the first lapse to cocaine use. Data were coded as right-censored for patients who were still abstinent at the end of monitoring or with whom the clinic lost contact. We also performed chi-squares for assessing differences in Day 90 functioning by cocaine frequency level and frequency changes compared to baseline.

Linear mixed models, with a random intercept for each subject, using the time-point as a 5 levels independent variable (“Baseline,” “Day 5,” “Day 30,” “Day 60,” “Day 90,”) were computed for each secondary outcome (CCQ, PSQI, BDI-II, SAS, GSI). To estimate the overall effect of treatment, group, and their interaction it was performed a type III analysis of variance with Satterthwaite's method for computing the denominator degrees of freedom of each F-test. We corrected multiple pairwise comparisons between time points using the Bonferroni method.

Thereafter, for examining the best predictor of change in cocaine frequency level we performed an ordinal logistic regression, testing the following predictors: group (ADHD/CocUD vs. CocUD), cocaine frequency level at baseline (abstinence vs. Low use vs. High use), age at the beginning of treatment, age at the first experience with cocaine, age at the time of addiction to cocaine, years of education, and baseline scores at CCQ, PSQI, BDI, SAS and GSI. We did not test for sex differences because most participants were male. To perform this analysis, we removed missing values in any of the predictors: the final sample consisted of 22 patients with ADHD in comorbidity with CocUD, and 156 CocUD patients.

Data were expressed as mean ± standard deviation (SD), unless otherwise specified; alpha was set at <0.05, two-tailed. All the analyses were performed using RStudio versions 1.2.5001 (51) with R version 3.6.1 (52) and the packages MASS (53), survival (54), lme4 (55), lmerTest (56), and emmeans (57).

## Results

### Patients Characteristics at Baseline

Demographic and clinical characteristics at baseline of the participants are presented in Table 1 divided by group. The sample of ADHD/CocUD consisted of 22 patients, 1 female and 21 males, aged between 25 and 53 (37.91 ± 8.71). The sample of CocUD-only consisted of 208 patients, 5 females 203 males, aged between 22 and 59 (37.67 ± 7.05). Table 1 shows the results of the independent sample *t*-test for assessing differences between groups. ADHD/CocUD patients were not significantly different compared to CocUD-only patients in demographic characteristics such as age, education, age at the first experience with cocaine, and age at the onset of addiction (all *p*s ≥ 0.37). Moreover, there were no significantly differences in craving for cocaine (CCQ, *p* = 0.82), self-perceived sleep quality (PSQI, *p* = 0.36), depression (BDI, *p* = 0.10), and anxiety (SAS, *p* = 0.06). However, a broader measure of clinical symptomatology such as the GSI, from SCL-90, revealed higher scores in ADHD/CocUD patients compared to CocUD-only patients (GSI, *p* = 0.03).

**Table 1:** Demographic and clinical characteristics of participants.

**Variables**	**ADHD/CocUD** **(*n* = 22)**	**CocUD-only** **(*n* = 208)**	***t***	**dF**	***P***
Age (years)	37.91 (8.71)	37.67 (7.05)	0.15	228	0.88
Gender (female/male)	1/21	5/203			
Education (years)	12.59 (3.5)	13 (3.21)	−0.91	228	0.57
Age at first experience (years)	20 (6.09)	21.27 (6.29)	0.23	228	0.37
Age at addiction (years)	29.64 (8.85)	29.83 (8.4)	−0.1	228	0.92
CCQ score at baseline	16.64 (13.11)	16.01 (11.91)	0.23	183	0.82
PSQI score at baseline	9.95 (3.95)	9.1 (4.14)	0.92	194	0.36
BDI-II score at baseline	22.05 (13.55)	17.99 (10.47)	1.66	209	0.10
SAS score at baseline	49.83 (10.19)	45.59 (10.13)	1.86	211	0.06
GSI score at baseline	69.75 (16.62)	62.61 (13.83)	2.24	210	0.03
**Cocaine use 30 days before baseline (% frequency level)**					
**Abstinence**	0	1	χ^2^ (2) = 2.16, *p* = 0.34		
**Low (1-4 uses)**	14	26			
**High (5+ uses)**	86	72			

*Data are presented as mean (standard deviation) unless otherwise specified. ADHD, attention-deficit/hyperactivity disorder; CocUD, cocaine use disorder; CCQ, cocaine craving questionnaire; PSQI, pittsburgh sleep quality index; BDI-II, beck depression inventory-II; SAS, self-rating anxiety scale; GSI, global severity index of the symptoms checklist 90 – Revised; Some percentages add up to slightly <100 due to rounding error*.

Regarding the cocaine use frequency level, most of the patients used 5 or more times in the 30 days before the beginning of treatment (ADHD/CocUD: 86%; CocUD-only: 72%). Only 1% of patients in the CocUD-only group was already abstinent at the beginning of treatment. A chi-square test of independence showed that there was no significant association between group and cocaine frequency level, χ^2^ (2) = 2.16, *p* = 0.34.

### Primary Outcome: Cocaine Use

**Figure 1:**
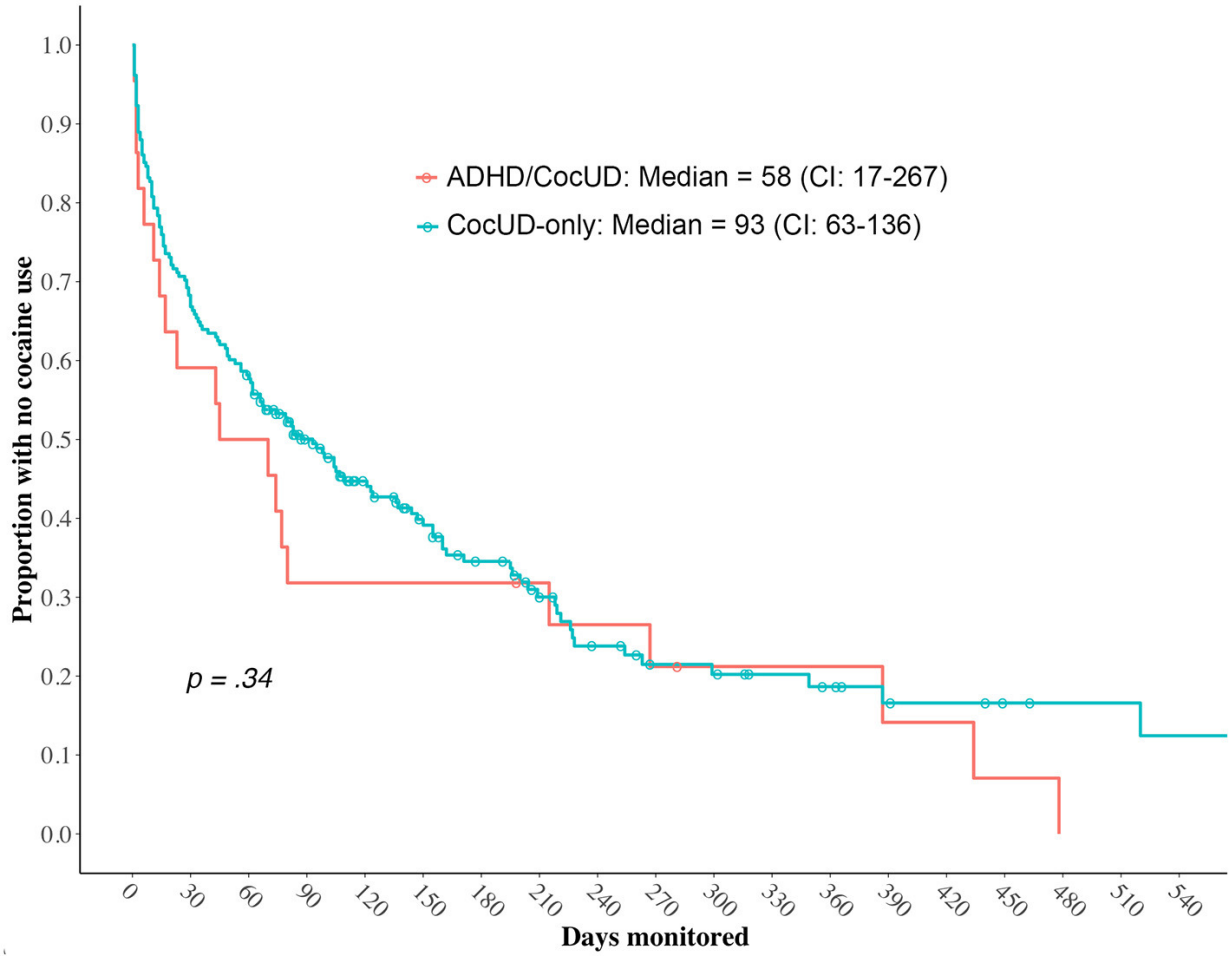
Time to the first resumption of cocaine in ADHD/CocUD and CocUD-only groups. ADHD, attention-deficit/hyperactivity disorder; CocUD, cocaine use disorder.

The Time to the first lapse is shown in Figure 1. The median time to the first use of cocaine use in the ADHD/CocUD group was 58 days (95% confidence interval: 17–267); in the CocUD-only group it was 93 days (95% confidence interval: 63–136). The difference between the two groups was not statistically significant (*p* = 0.34).

**Figure 2:**
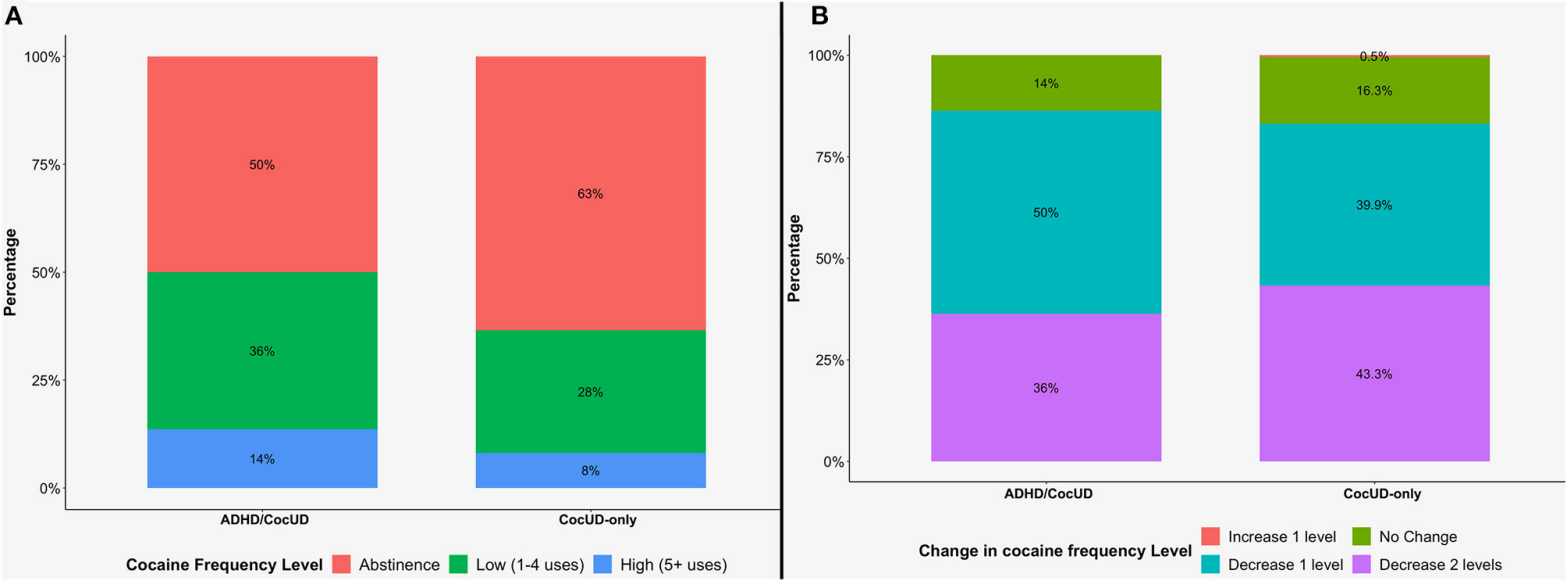
Distribution of patients according to cocaine frequency level at Day 90 **(A)**, and change in cocaine frequency level in comparison to baseline **(B)**. ADHD, attention-deficit/hyperactivity disorder; CocUD, cocaine use disorder.

At the end of the standard protocol of treatment (Day 90), based on the cocaine use during the 30 days before the assessment, we specified three frequency levels as we did at baseline (Figure 2A). The proportion of abstinent patients significantly increased over time in both the ADHD/CocUD group [χ^2^ (2) = 24.9, *p* < 0.001] and the CocUD-only group [χ^2^ (2) = 229.33, *p* < 0.001]: respectively 50 and 63% of patients were abstinent during the 30 days prior to Day 90. There were no differences between groups [χ^2^ (2) = 1.69, *p* = 0.42]. Concerning the variation in cocaine frequency level from baseline to Day 90, 86% of ADHD/CocUD and 82% of CocUD-only patients reported an improvement (decrease one or two levels) (Figure 2B). Again, the chi-square test of independence showed that there was no significant association between groups and the variation in cocaine frequency level [χ^2^ (3) = 0.91, *p* = 0.82].

### Secondary Outcome: Changes in Craving, Sleep, Depression, and Anxiety

The second goal of our analyses was to investigate changes in clinical outcomes over time and whether there were differences between ADHD/CocUD and CocUD-only patients undergoing rTMS over the L-DLPFC. Using type III analyses of variance, we tested the main effect of *Time* and *Group* and their interaction in each linear mixed model for the different clinical outcomes.

CCQ scores significantly improved at each timepoint after the first week of rTMS treatment [*F*_(4, 638)_ = 50.35, *p* < 0.001]. There were no differences between groups and there was not a significant effect of the *Time* × *Group* interaction [*F*_(4, 638)_ = 0.43, *p* = 0.78]. Pairwise comparisons showed that CCQ scores at Day 5 were significantly lower than those at baseline in both the ADHD/CocUD group (Day 5: 5.59 ± 7.53; Baseline: 16.64 ± 13.11; *p* < 0.001), and CocUD-only group (Day 5: 3.81 ± 4.95; Baseline: 16.01 ± 11.9; *p* < 0.001). This improvement was maintained through the three subsequence time points in both the groups: ADHD/CocUD Day 30 (6.64 ± 8.17; *p* < 0.001), CocUD-only Day 30 (3.12 ± 5.67; *p* < 0.001), ADHD/CocUD Day 60 (5.55 ± 8.54; *p* < 0.001), CocUD-only Day 60 (3.62 ± 7.25; *p* < 0.001), ADHD/CocUD Day 90 (4.71 ± 5.46; *p* < 0.001), CocUD-only Day 90 (3.19 ± 5.45; *p* < 0.001).

Like craving, we observed a significant reduction over time of sleep disturbances and affective symptoms as reflected by the significant main effect of *Time* in each linear mixed model: PSQI [*F*_(4, 682)_ = 28.99, *p* < 0.001], BDI-II [*F*_(3, 518)_ = 101.88, *p* < 0.001], SAS [*F*_(4, 676)_ = 43.87, *p* < 0.001], and GSI [*F*_(4, 735)_ = 92.73, *p* < 0.001]. Also, for all these measures it was observed a main effect of *Group*: PSQI [*F*_(1, 204)_ = 8.01, *p* < 0.01], BDI-II [*F*_(1, 200)_ = 4.48, *p* < 0.05], SAS [*F*_(1, 217)_ = 13.13, *p* < 0.001], and GSI [*F*_(1, 220)_ = 11.26, *p* < 0.001]. Pairwise comparison allowed to highlight the differences between groups at the different time points. As previously observed, and here confirmed, at baseline groups were significantly different only for GSI scores [*t*_(566)_ = 3.03, *adjusted p* = 0.01]. After the first week of treatment both the groups significantly improved in all the scores, and pairwise comparison showed no significant differences for any of the clinical measures, neither at GSI [*t*_(566)_ = 2.37, *adjusted p* = 0.09]. At Day 30, pairwise comparison highlighted significant differences between groups for PSQI [*t*_(672)_ = 2.99, adjusted *p* = 0.01], SAS [*t*_(633)_ = 2.77, *adjusted p* = 0.02], and GSI scores [*t*_(709)_ = 2.59, *adjusted p* = 0.04]. Other comparison showed that PSQI scores at Day 30 in ADHD/CocUD patient were no longer different from baseline [*t*_(678)_ = 2.28, *adjusted p* = 0.26]. However, in all the other cases the scores at Day 30 were still significantly lower than those at baseline in both groups. At Day 60 and Day 90 the differences between groups returned to be not significant for all the clinical measure but SAS [Day 60: *t*_(585)_ = 2.93, *adjusted p* = 0.02; Day 90 *t*_(628)_ = 3.06, *adjusted p* = 0.01]. Also, PSQI score of ADHD/CocUD patients improved and turned again to be significantly lower than those at baseline [Day 60: *t*_(683)_ = 3.74, *adjusted p* = 0.002; Day 90 *t*_(679)_ = 3.77, *adjusted p* = 0.001].

For none of the clinical outcomes significant *Time* × *Group* interactions (all *p*s ≥ 0.27) were observed.

### Best Predictor of Change in Cocaine Frequency Level

In a separate model, we examined the best predictor of change in cocaine frequency level from baseline to day 90 performing an ordinal logistic regression. The results are summarized in Table 2. Above all the predictors, only the cocaine frequency level at baseline and the CCQ score reached the defined alpha level (α = 0.05). Higher cocaine frequency level at baseline was associated with higher odds of moving from *no change* to *decrease one level* or *decrease two levels* (OR = 9.76; 95% CI: 4.61–21.77). Also, for a one-unit increase in CCQ score, the odds of moving from *no change* to *decrease one level* or *decrease two levels* were 4% less, given that the other variables in the model are held constant.

**Table 2:** Coefficient table of the ordinal logistic regression for examining the best predictor of change in cocaine frequency level.

**Variables**	**Value**	**Std. Error**	***t*-value**	***P-*value**
Group	0.508	0.468	1.085	0.27
Cocaine frequency level at baseline	2.279	0.395	5.774	<0.001^**^
Age	0.027	0.028	0.973	0.33
Education	−0.038	0.048	−0.798	0.42
Age at first experience	0.003	0.033	0.103	0.91
Age at addiction	0.010	0.027	0.392	0.69
CCQ score at baseline	−0.035	0.015	−2.302	0.02^*^
PSQI score at baseline	−0.060	0.048	−1.241	0.21
BDI-II score at baseline	0.013	0.025	0.533	0.59
SAS score at baseline	0.002	0.026	0.084	0.93
GSI score at baseline	0.001	0.018	0.033	0.97

* p < 0.05;

** *p < 0.001*.

*CCQ, cocaine craving questionnaire; PSQI, Pittsburgh sleep quality index; BDI-II, beck depression inventory-II; SAS, self-rating anxiety scale; GSI, global severity index of the symptoms checklist 90 – revised*.

### Safety

None of these 230 patients reported any serious adverse event during the study. There were no seizures, syncopes, neurological complications, or subjective complaints about memory or concentration impairment limiting the treatment and no patient discontinued treatment prematurely due to intolerable stimulation, pain, or other adverse effects such as headache, vertigo, or fatigue.

## Discussion

The main aim of the present study was to determine whether attention deficit hyperactivity disorder (ADHD) comorbidity among patients with cocaine addiction is associated with higher clinical symptomatology or less successful results of rTMS treatment.

In our sample the prevalence of ADHD was 9.5%, which is very close to what was found in other populations of cocaine abusers (9), and higher than the one reported in the Italian population (2.8%) (58). In opposite to already published studies and meta-analyses (5, 15, 16), in our cohort cocaine abusers with adult ADHD, compared to those without such comorbidity, were not younger at the clinical admission and did not report an earlier onset of cocaine abuse or a more frequent use in the 30-days before treatment. Moreover, they did not report worse depressive symptomatology, self-perceived quality of sleep, or anxiety as assessed by BDI-II, PSQI, and SAS. At baseline, the only clinical measure which was significantly different between the two groups was the Global Severity Index, indicating a generical status with severe symptoms. The lack of differences between groups may be due to an uncontrolled bias regarding the intrinsic characteristics of the patients who voluntarily refers to the specialty outpatient private clinic in which data were collected. They may have a higher socio-economic status or higher level of education compared to the generic population of cocaine abusers. These elements may flatten the differences found in the already published studies. Further studies are needed to test this hypothesis.

Several studies suggested that psychiatric comorbidity could play a role in determining a worse prognosis (5, 17, 18). Thus, we predicted that co-occurring ADHD would have a negative impact on the outcome of treatment (e.g., cocaine use). In our study, we adopted a harm reduction approach already validated for alcohol and cocaine consumption (44, 45). As reported by other groups, other than abstinence, a reduction in cocaine frequency by the end of treatment might be meaningful for a sustained clinical benefit up to 1 year following treatment (45). Surprisingly, our findings did not replicate the negative prognostic effect: concerning the variation in cocaine frequency level from baseline to Day 90, 86% of ADHD/CocUD and 82% of CocUD-only patients reported an improvement (decrease one or two levels) with no significant differences between groups. Both groups also showed an overall significant improvement of other accompanying symptoms, including depression and perceived sleep quality. On Day 90 there were no differences between groups in none measure, except for SAS scores. Indeed, patients with ADHD comorbidity showed higher anxiety levels compared to CocUD-only patients at Day 60 and Day 90. However, the mean SAS score in ADHD patients was above the clinical level set to 45, indicating a normal range of anxiety in both groups.

In our sample of ADHD/CocUD patients, 19 out of 22 subjects were pharmacologically treated with atomoxetine, and all received an rTMS treatment in addition to a conventional psychosocial intervention. This integrative multidimensional approach could account for the positive outcome observed in the ADHD/CocUD patient population, that did not differ from the CocUD-only group. However, despite atomoxetine treatment has been associated with clinical improvements in quality of life and executive functions in subjects with ADHD (59), a randomized double-blind placebo-controlled study failed to provide evidence supporting the utility of atomoxetine in treating cocaine dependence (60, 61). Moreover, it has been reported that atomoxetine increases extracellular levels of DA in prefrontal cortex, but not in the striatum and nucleus accumbens (62–65). The rTMS neuromodulatory effect within the reward circuitry may induce significant changes within the dysfunctional dopaminergic signaling underlying ADHD pathophysiology. Functional imaging studies showed a significant reduction dopamine transporter (DAT) and D_2_/D_3_ receptors within the reward/motivation brain areas in both ADHD and CocUD patients compared to healthy subjects (21, 22, 66, 67). The rTMS protocol over the left DLPFC might restore the aberrant dopaminergic signaling through the dopamine release induced in the caudate nucleus, cingulate cortex, and other regions of the dopamine pathway (68, 69) in both ADHD and addiction conditions. Thus, the modulation of dopamine signaling and the effects on executive functioning due to the rTMS treatment, rather than atomoxetine, may lead to the significant clinical effects we observed indiscriminately in both ADHD/CocUD and CocUD-only patients. This may open a new view in the investigation of the therapeutic effect of high-frequency stimulation on ADHD symptoms. Indeed, conflicting results have been reported regarding the use of rTMS as an effective tool for ADHD treatment (40, 41, 70–72). However, none of these studies stimulated the left DLPFC and further studies are needed to examine his role.

Another aim of our study was to explore the better predictor of treatment outcome. Specifically, we examined the best predictor of change in cocaine frequency level from baseline to day 90 performing an ordinal logistic regression. Above all the predictors, only the cocaine frequency level at baseline and the craving were significant. In previous studies, both of these variables were the most important predictors of successful detoxification from cocaine (73–77). Our results extend these findings to the context of an rTMS treatment. Again, there were no differences between groups: having ADHD in comorbidity is not related to a decreased odd of improvement.

To our knowledge, this is the first study comparing the demographic characterization and rTMS clinical improvements of patients with a dual diagnosis of ADHD and CocUD against CocUD-only patients. Cocaine use and common self-reported withdrawal/abstinence symptoms appear to benefit from rTMS treatment with no differences between groups.

We are aware of the limitations of the naturalistic clinical setting in which our cohort of patients received an rTMS treatment. Considering the absence of a control group or a sham-controlled double-blind design, we cannot rule out a possible placebo effect. Moreover, the unbalanced samples and the lack of a priori power analysis could have influenced the final outcome. Future studies using a more standardized approach are needed to further investigate these preliminary results.

## Data Availability Statement

The dataset used in this study is not publicly available due to the sensitive and personal nature of the information included. However, the corresponding author is willing to respond to any reasonable requests for de-identified data.

## Ethics Statement

The protocol, limited to the retrospective chart review, was reviewed and approved by Ethical Committee for the Psychological Research, Departments of Psychology, University of Padua (Protocol no. 3185, code 82F319362FA08A4C9498620BF072CB72). The patients provided their written informed consent to participate in this study.

## Author Contributions

SC: data curation, methodology, formal analysis, and writing the original draft. GM and LG: conceptualization, supervision, review and editing original draft. LGP and DC: methodology and data curation. MS, NC, and AT: review and editing the original draft and designed the study. All authors contributed to the article and approved the submitted version.

## Conflict of Interest

The authors declare that the research was conducted in the absence of any commercial or financial relationships that could be construed as a potential conflict of interest.
