# Supplementary material for: A Retrospective Comparative Study in Patients With Cocaine Use Disorder Comorbid With Attention Deficit Hyperactivity Disorder Undergoing an rTMS Protocol Treatment
Source: Front Psychiatry. 2021 Mar 25;12:659527. doi: 10.3389/fpsyt.2021.659527 (PMC8026860; doi:10.3389/fpsyt.2021.659527)
Supplement: Supplementary file 1 [file Data_Sheet_1.PDF]

Table 1. Post hoc analysis for t tests.

Variables	t	dF	*p*	*n ADHD*	*n CocUD-only*	*d*	*Power*
Age (years)	0.15	228	.88	22	208	.03	.05
Education (years)	-0.91	228	.57	22	208	.13	.09
Age at first experience (years)	0.23	228	.37	22	208	.20	.15
Age at addiction (years)	-0.1	228	.92	22	208	.02	.05
CCQ score at baseline	0.23	183	.82	22	163	.05	.06
PSQI score at baseline	0.92	194	.36	22	174	.20	.15
BDI-II score at baseline	1.66	209	.10	22	189	.37	.38
SAS score at baseline	1.86	211	.06	22	191	.42	.46
GSI score at baseline	2.24	210	.03	22	190	.51	.61
